# Harvesting the Casualties of War: *Macrogerodonia peruviana* Rove Beetles Prey Exclusively Upon Wounded *Trigona* spp. Stingless Bees (Coleoptera: Staphylinidae; Hymenoptera: Apidae)

**DOI:** 10.1002/ece3.73850

**Published:** 2026-06-18

**Authors:** Erin Rivera, Alejandro Lopera, Raider Castro, Sam Pottie, Adrian Forsyth

**Affiliations:** ^1^ Andes Amazon Fund Washington DC USA; ^2^ School of Geography and the Environment University of Oxford Oxford UK; ^3^ Manu Biological Station Pillcopata Cusco Peru; ^4^ Universidad Ricardo Palma Lima Peru

**Keywords:** *Macrogerodonia*, necrophagy, predation, rainforest, *Staphylinidae*, stingless bees, *Trigona*

## Abstract

Social insects represent a major component of tropical forest biomass, yet the ecological fate of their necromass remains poorly understood. Stingless bees (Apidae: Meliponini), particularly species in the genus *Trigona*, frequently engage in aggressive, sometimes lethal conflicts while competing for nutrient‐rich resources such as carrion. Here we report a previously undescribed trophic interaction in which rove beetles of the species *Macrogerodonia peruviana* (Staphylinidae) prey exclusively on wounded or recently killed *Trigona* workers. Field observations and cafeteria choice experiments showed that beetles consistently preyed on injured *Trigona* spp. bees, ignoring carrion and alternative invertebrates, including flies, grasshoppers, and other stingless bee genera. This behavior was observed at different sites in Peru and Costa Rica, suggesting this interaction is geographically widespread. Our observations indicate a previously undescribed, highly specialized trophic interaction linking social insect warfare to predator specialization, emphasizing the ecological significance of social insect necromass in structuring tropical forest food webs.

## Graphical abstract

We report a previously undescribed trophic interaction in which *Macrogerodonia peruviana* rove beetles prey exclusively on wounded or recently killed *Trigona* stingless bees generated during aggressive conflicts at carrion. Field observations and choice experiments showed that beetles selectively targeted injured *Trigona* while ignoring carrion and other invertebrates. This specialized interaction links social insect warfare to predator specialization and documents a previously unrecognized pathway in tropical forest food webs.

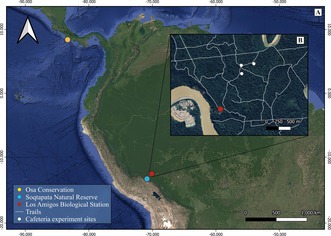

Social insects comprise a large proportion of the animal biomass in many forest ecosystems (Schultheiss et al. 2022; Rosenberg et al. 2023; Basset et al. 2023). Despite this, the fate of social insect necromass remains poorly understood, although it may play a substantial role in nutrient cycling, food webs, and other aspects of tropical forest functioning (Vasconcellos and Moura 2010; Palace et al. 2012; Benbow et al. 2019; Tuma et al. 2022; Silva et al. 2021; Griffiths et al. 2019). Stingless bees (Apidae: Meliponini) are among the most ecologically important social insects in tropical forests, with colonies reaching large sizes, on the order of 100,000 workers in the case of *Trigona amazonica* (reviewed in Grüter 2021). Their high colony densities and generalized foraging behavior often make them the dominant pollinators of both wild plants and crops, key mediators of plant reproduction, and major contributors to forest resilience (Roubik 1982; Heard 1999; Slaa et al. 2006; Giannini et al. 2015).

While stingless bees primarily forage for nectar, pollen, and resins, many species also exploit carrion and other nutrient‐dense resources (Roubik 1982; Figueroa et al. 2021; Dorian and Bonoan 2020). In these contexts, competition can be intense, with some species engaging in physical combat and even exhibiting “suicidal biting” behavior (Cunningham et al. 2014; Grüter et al. 2016); similarly, some *Trigona* species employ group recruitment and aggressive behaviors as foraging strategies (Johnson and Hubbell 1974). In particular, the genus *Trigona*, which includes several carrion‐feeding species, is often highly aggressive (reviewed in Grüter 2021). Johnson and Hubbell (1974) documented prolonged battles between conspecific *Trigona* colonies that lasted up to 2 days. Despite these well‐documented conflicts, the ecological consequences of stingless bee interactions, particularly the fate of injured and dead individuals and their role within food webs, remain poorly understood. Here we report that *Macrogerodonia peruviana* rove beetles prey exclusively upon wounded and recently killed stingless bees in the genus *Trigona*.

During field studies of the necrophagous dung beetle *Coprophanaeus lancifer* at the Los Amigos Biological Station in southeastern Peru (−12.3257 S, −70.3819 W), we repeatedly observed *Trigona* spp. bees visiting carrion baits. At these sites, bees frequently fought, producing injured (e.g., unable to walk or fly but still capable of movement) and dead workers that accumulated around the baits. These injured bees in turn attracted small, agile rove beetles (Staphylinidae). Upon landing near an injured bee, the beetles quickly bit it and dragged it away from the bait beneath the leaf litter. This observation raised the question of whether the beetles were simply exploiting injured prey opportunistically or were instead specifically associated with wounded *Trigona* workers. To address this, we conducted a series of choice experiments alongside additional field observations.

First, to determine whether *Macrogerodonia* sp. beetles were attracted to wounded bees themselves, as opposed to carrion, we presented two treatments: (1) carrion alone and (2) wounded or recently killed *Trigona* workers alone. Beetles were attracted only to wounded *Trigona* workers and did not arrive at carrion presented alone. Specimens of these beetles were subsequently collected and identified by specialists as *Macrogerodonia peruviana* (Bernhauer 1908) based on comparison with type material.

Next, to assess whether the beetles were specialized on wounded *Trigona* workers or exploited other injured invertebrates at carrion, we conducted a series of cafeteria‐style choice experiments using insect taxa commonly attracted to carrion at three different locations in terra firme forest at the Los Amigos Biological Station (Figure 1B). At each site, we established experimental arenas by clearing small areas of forest floor (approximately 1–2 m in diameter) of leaf litter to allow unobstructed observation. Within each arena, three large dry leaves were placed approximately 20 cm apart and used as presentation surfaces for prey items. Of these three leaves, one contained wounded or recently killed *Trigona* workers (either *T. truculenta* or *T. williana* ), while the other two contained alternative injured prey, each leaf presenting a single taxon. The alternative prey taxa were drawn from a pool including Mesembrinellidae and Phoridae flies, clown grasshoppers (*Paramastax nigra*), and the stingless bee *Partamona vicina* . The specific combination of alternative taxa varied among trials, such that not all prey types were presented simultaneously in a single replicate. Individuals were experimentally injured immediately prior to trials by gentle compression, rendering them unable to fly or walk while remaining alive and capable of movement (e.g., leg or body motion); the same procedure was applied consistently across all taxa. All prey were then placed on the leaves at the same time to ensure that beetles encountered all options under comparable conditions. Each arena was observed for 20‐min periods, during which we recorded the prey item selected by arriving beetles. This design was replicated five times at each of the three sites, for a total of 15 trials. Across all trials, *M. peruviana* consistently selected wounded *Trigona* workers and did not attack any alternative prey, including *Partamona vicina* .

**FIGURE 1:**
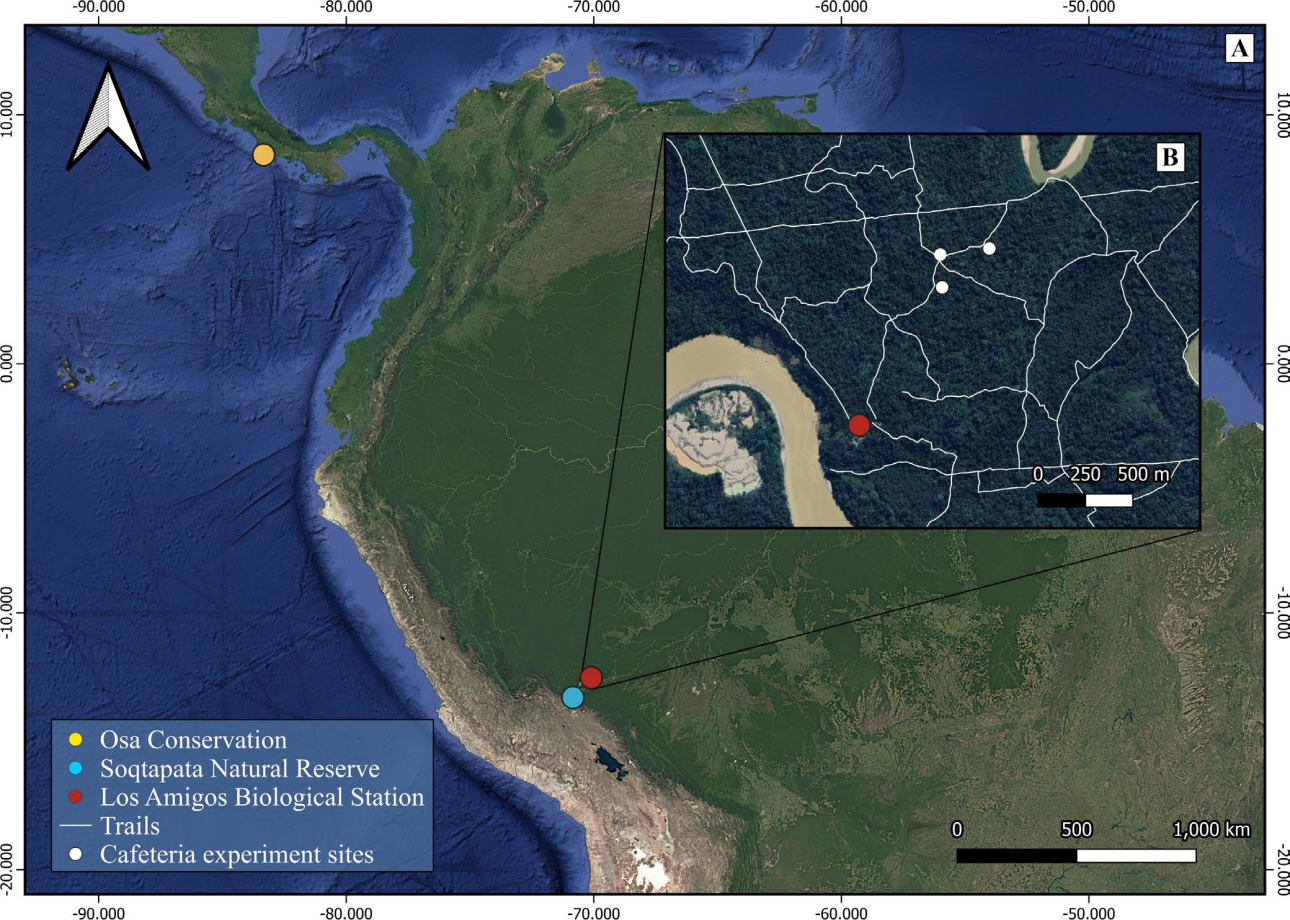
(A) Map of Neotropical locations where interactions between *Macrogerodonia peruviana* rove beetles and wounded *Trigona* spp. stingless bees were observed, including primary study sites in southeastern Peru (Los Amigos Biological Station) and additional opportunistic observations in Peru (Soqtapata Nature Reserve) and Costa Rica (Osa Conservation). (B) Schematic of cafeteria‐style choice experiment conducted at Los Amigos Biological Station, showing experimental arenas with three prey presentations: wounded *Trigona* workers and alternative injured invertebrate taxa placed on separated leaves to assess prey preference by *M. peruviana* .

To place these interactions in a broader ecological context, we conducted direct field observations using Reconyx cameras. These were set in close‐focus and time‐lapse recording to monitor insect activity and interactions at different carrion baits. These included rotten chicken, fish, and guinea pigs placed in the leaf litter, as well as a pig carcass deployed by another research group studying vultures (Grootaers et al. 2025). Across these observations, carrion bait attracted a diverse assemblage of insects, including Phoridae and Mesembrinellidae flies, clown grasshoppers ( *P. nigra* ), Euglossine bees, ants, Epiponine social wasps such as *Stelopolybia* spp., cockroaches, geometrid moths, and nymphalid butterflies. Stingless bees were frequent and often abundant visitors, and in some cases, such as at the pig carcass, arrived in large numbers and engaged in intense fighting that produced several injured individuals. Despite the availability of multiple potential prey types, *M. peruviana* was observed attacking only wounded *Trigona* workers and did not attack any of the other taxa present. Phoridae flies are known to exploit injured social insects as food sources and oviposition sites (Segura and Brown 2014; Witte et al. 2010; Sharma et al. 2011; Chen and Fadamiro 2018). Their frequent presence on injured bees suggests that wounded *Trigona* workers may represent a shared and potentially contested resource for multiple taxa. Close observation of these interactions revealed a highly stereotyped sequence of attack by *M. peruviana* . Individuals approached by direct, linear flight, typically landing within 10 cm of a bee. Upon contact, beetles rapidly drummed their antennae over the bee's body before quickly biting and grasping a bee appendage, most often a limb. During this interaction, the abdomen was held elevated and moved continuously. The beetle then dragged the bee away from the bait in a roughly straight line and, after moving approximately 1 m, pulled it beneath the leaf litter. In some cases, multiple beetles converged on a single injured bee, with up to six individuals observed competing for control (Video S1).

To characterize post‐capture feeding behavior, we conducted feeding observations using six beetles collected at carrion baits and maintained individually in plastic containers with soil and leaf litter. Each individual was provided with recently killed *Trigona* workers and monitored at multiple time points over a 24‐h period. Feeding most commonly began at the head, which was the first body region targeted in five out of six cases; in one instance, a beetle also fed on the thorax and abdomen. These observations indicate that *M. peruviana* consumes *Trigona* workers following capture, exhibiting a consistent pattern of tissue preference.

Additional opportunistic observations were recorded at two other Neotropical localities (Figure 1A), although these records were not part of the standardized choice experiments. At Soqtapata Natural Reserve, a montane mesic forest locality in the Peruvian Andes at approximately 1400 m elevation, *Trigona* bees aggregated in a researcher's sweat‐soaked backpack, presumably attracted by sodium salts. Aggressive interactions among the bees produced injured individuals, after which a *Macrogerodonia* beetle was observed arriving at the aggregation (Video S2). Following this observation, additional non‐standardized baiting trials were conducted at Soqtapata using tuna bait and urine to attract and aggregate *Trigona* bees. These trials also produced aggressive interactions among workers and allowed observations of rove beetles scavenging or preying upon injured individuals. The beetles exhibited the same general attack and prey‐handling sequence observed at Los Amigos Biological Station, including direct approach, antennal contact, seizing the bee, and dragging it away from the aggregation into the leaf litter. A comparable observation was made at Osa Conservation, on the Osa Peninsula, Costa Rica, where *Macrogerodonia* beetles were attracted to injured or recently killed *Trigona* workers. Although these observations were opportunistic and not replicated quantitatively, they suggest that similar interactions between *Macrogerodonia* beetles and injured *Trigona* workers may occur beyond the main study site.

These observations suggest that wounded *Trigona* workers may constitute a predictable, spatially concentrated resource generated by aggressive foraging interactions (Yang et al. 2008). In tropical ecosystems, nutrient‐rich resources such as carrion and sodium sources are often ephemeral and highly contested (Chaboteaux et al. 2025). Several *Trigona* species recruit strongly to such resources and defend them aggressively, sometimes engaging in physical conflicts that injure or kill workers. These conflicts may repeatedly create localized prey patches for *M. peruviana*. By contrast, stingless bee genera that forage less aggressively occur at lower densities at these resources or generate fewer injured workers during competition may provide less predictable opportunities for beetle predation. Thus, the selectivity observed may arise because of the distinctive way in which aggressive *Trigona* foraging behavior generates accessible injured prey.

The injuries observed in this study appeared functionally severe: affected bees were unable to fly or walk normally, although some individuals remained alive and capable of limited movement. Such workers were therefore unlikely to resume normal foraging, and their accumulation around contested resources may represent an underappreciated cost of aggressive resource defense. We did not observe nestmates rescuing, retrieving, or otherwise caring for injured workers. This contrasts with rescue and wound‐care behaviors described in other social insects such as some ants, where injured workers may be carried back to the nest and treated by nestmates (Frank et al. 2017). This might indicate that the fate of injured social insects may vary strongly among taxa, depending on whether injured workers remain valuable to the colony, whether they can be retrieved, and whether predators or parasitoids exploit them before nestmates can respond. The interaction reported here may therefore link social insect conflict to predator specialization.

Aggressive competition among *Trigona* bee workers produces casualties, and these injured individuals appear to be targeted by *M. peruviana* beetles using a stereotyped attack and prey‐handling sequence. Similar systems are known in phorid flies associated with social insects, where parasitoids or scavenging flies exploit injured hosts and may use host alarm cues or injury‐associated signals to locate vulnerable individuals (Segura and Brown 2014; Sharma et al. 2011; Witte et al. 2010). The presence of both beetles and flies at injured bees raises the possibility that these casualties are readily detectable to multiple consumers, although the cues involved and their relative importance remain unclear and warrant further investigation.

From an evolutionary perspective, repeated access to injured *Trigona* workers could favor a consistent prey‐handling strategy adapted to exploiting vulnerable but potentially contested prey, resulting in behavioral specialization of *M. peruviana* . Future work should quantify the frequency of injuries at contested resources, compare beetle responses to different stingless bee genera and injury states, and test whether beetles use chemical cues associated with wounded bees, alarm signaling, or resource aggregations. Such studies would clarify whether *M. peruviana* specialization is driven primarily by prey identity, injury‐related cues, or the predictable ecological context created by aggressive *Trigona* foraging.

## Author Contributions

**Erin Rivera:** investigation (equal), methodology (equal), visualization (lead), writing – original draft (equal), writing – review and editing (lead). **Alejandro Lopera:** conceptualization (equal), investigation (equal), methodology (equal), writing – original draft (supporting). **Raider Castro:** formal analysis (equal), investigation (supporting). **Sam Pottie:** investigation (equal), writing – review and editing (supporting). **Adrian Forsyth:** conceptualization (lead), funding acquisition (lead), investigation (equal), methodology (lead), writing – original draft (lead), writing – review and editing (supporting).

## Funding

This work was supported by funding from the NGO Climate Corridors.

## Conflicts of Interest

The authors declare no conflicts of interest.

## Supporting information


**Video S1:** Sequence of videos demonstrating the predatory interaction between *Macrogerodonia peruviana* and injured *Trigona* sp. stingless bees. First, aggressive interactions between *Trigona* bees that resulted in injured individuals, followed by the arrival and aggregation of *Macrogerodonia peruviana* beetles. Multiple beetles were observed converging on injured bees, and close‐up footage shows beetles interacting with injured *Trigona* individuals alongside phorid flies. In the final sequence, a *Macrogerodonia peruviana* beetle drags an injured *Trigona* bee away from the aggregation, presumably for consumption.

**Video S2:** Opportunistic observation of *Macrogerodonia* sp. attracted to a *Trigona* sp. aggregation in Soqtapata Natural Reserve, Peru.

## Data Availability

The data supporting the findings of this study are provided as Supporting Information.
